# EBDC: An Energy-Balanced Data Collection Mechanism Using a Mobile Data Collector in WSNs

**DOI:** 10.3390/s120505850

**Published:** 2012-05-07

**Authors:** Chih-Yung Chang, Chih-Yu Lin, Chin-Hwa Kuo

**Affiliations:** Department of Computer Science and Information Engineering, Tamkang University, No. 151, Yingzhuan Road, Tamsui District, New Taipei City 25137, Taiwan; E-Mails: cylin@wireless.cs.tku.edu.tw (C.-Y.L.); chkuo@mail.tku.edu.tw (C.-H.K.)

**Keywords:** wireless sensor networks, data collection, mobile sink, energy balancing

## Abstract

The data collection problem is one of the most important issues in Wireless Sensor Networks (WSNs). Constructing a tree from all sensor nodes to the sink node is the simplest way, but this raises the problem of energy unbalance since the sensors closer to the sink node would have much higher workloads from relaying data. To cope with the energy unbalance problem, a number of mobile-sink mechanisms have been proposed in recent years. This paper proposes an Energy-Balanced Data Collection mechanism, called *EBDC*, which determines the trajectory of a mobile data collector (or mobile sink) such that the data-relaying workloads of all sensors can be totally balanced. Theoretical analysis and performance evaluation reveal that the proposed *EBDC* mechanism outperforms the existing approaches in terms of network lifetime and the degree of energy balancing.

## 1. Introduction

Wireless Sensor Networks (WSNs) have many potential applications, which include environmental monitoring, tracking, healthcare, surveillance, smart homes and so forth [1–4]. Since sensors are battery powered, prolonging the network lifetime of WSNs is crucial for the usage of sensors in this wide range of applications. Communication is one of the major sources of energy consumption. With limited transmission range, sensors typically deliver their readings to the sink in a multi-hop manner. This behavior will raise the problem of unbalanced energy since the sensors closer to the sink have heavier data-relaying workloads and thus exhaust their energies much faster than the more distant sensors [5–7]. As a result, the network will be partitioned and hence the sink can become unreachable by other sensors.

Instead of constructing a data collection tree from a fixed sink to all sensors, a number of studies [8–11] have employed a mobile data collector (or mobile sink) moving along some predefined trajectory to migrate the data-relaying workload from one sensor to another. In [8] and [9] a trajectory which enables the mobile sink to directly communicate with sensors was constructed. However, the length of trajectory increases with the size of the monitoring region. This is because the constructed trajectory has to pass through the transmission range of each sensor. As a result, sensors have to wait for a long time to be visited by mobile sink again, leading to a long visit latency.

Zhao *et al.* [10] selected some sensors as the tree roots and then constructed a tree from all the other sensors to each root. By visiting the selected roots in turn, a mobile sink can collect the readings generated by all sensors based on the constructed tree in a multi-hop manner. Compared to studies [8] and [9], the scheme proposed in [10] significantly reduces the trajectory length of the mobile sink. Nevertheless, the data-relaying workloads of roots are higher than those of the other sensors, resulting in an energy-unbalanced problem.

**Figure 1.**
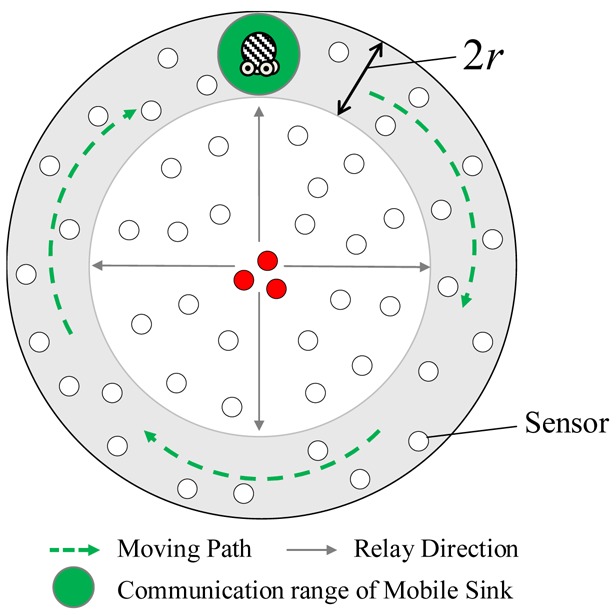
The red nodes and boundary sensors are energy-unbalanced.

Alsalih *et al.* [11] considered a circular monitoring region. All sensors are assumed to be uniformly deployed over the monitoring region. As shown in Figure 1, the mobile sink whose transmission range is *r* moves along the boundary of the monitoring region to collect readings. The sensors located at the boundary, called *boundary sensors*, can be visited by mobile sink while the remaining sensors have to deliver their readings to the mobile sink in a multi-hop manner due to the limited transmission range.

To forward the collected readings to the sink, the boundary sensors will receive and store the readings and then wait for mobile sink to pass through their transmission ranges. However, sensors closer to the center of the monitoring region would have much fewer data-relaying workloads than the boundary sensors. For example, as shown in Figure 1, each of red nodes only needs to deliver their own readings to their neighbors without any data-relaying workloads. Consequently, the boundary sensors and the red nodes have different energy consumptions, leading to an energy-unbalance problem. This paper proposes an Energy-Balanced Data Collection mechanism, called *EBDC*, which determines a trajectory such that the data-relaying workloads of all sensors can be totally balanced. Similar to the network environment of study [11], this paper considers a circular monitoring region which has been geographically partitioned into a number of circular tracks. To balance the data-relaying workloads, the mobile sink moves along different tracks with predefined sweep repetitions. At any given time, each sensor is able to derive the track where the mobile sink is visiting currently. Therefore, each sensor can send its reading to the appropriate neighbor such that the reading can reach the mobile sink in a multi-hop manner.

Furthermore, the proposed *EBDC* mechanism can be applied to a wide range of applications. For example, in an environmental monitoring application, a large number of sensors can be randomly deployed over a monitoring region to monitor temperature, humidity or air quality. Instead of reporting data frequently, sensors in such application only need to report their readings to the sink periodically. Hence, this scenario motivates us to use a mobile sink to collect data.

The remaining part of this paper is organized as follows: Section 2 presents the network environment and problem formulations of our approach, while Section 3 presents the details of the proposed *EBDC* mechanism. Sections 4 and 5 investigate the theoretical analysis and the performance of the *EBDC* mechanism, respectively. Finally, the conclusions of this paper are given in Section 6.

## 2. Network Environment and Problem Formulation

This section initially introduces the network environment and the assumptions of the given WSN. Then, the notations used in this section and the problem formulations of our approach are proposed.

### 2.1. Network Environment

**Figure 2.**
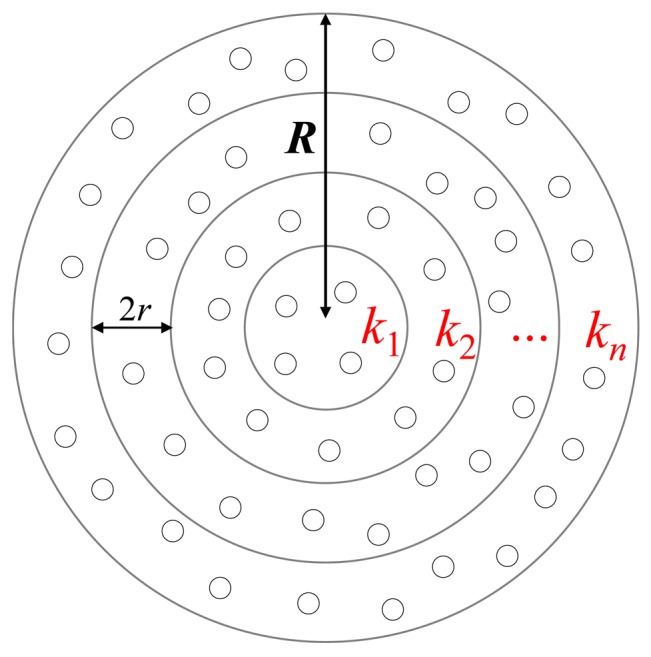
Monitoring region *M* is geographically partitioned into *n* = *R*/2*r* circular tracks which are traversed by mobile sink.

Given a circular monitoring region *M*, this paper assumes that all sensors are randomly deployed in *M*. Let the radius of the monitoring region *M* be *R*. Let the transmission ranges of the mobile sink and all sensors be identical and equal to *r*. Herein, we assume that the mobile sink and all sensors are aware of their own location information by GPS or other location support system and each sensor also knows its neighbors' locations. As shown in Figure 2, to balance the data-relaying workloads of all sensors, the monitoring region *M* is geographically partitioned into *n* = *R*/2*r* circular tracks where the thickness of each track is 2*r*. Let *K* = {*k_i_* | 1 ≤ *i* ≤ *n*} and *S* = {*s_i_* | 1 ≤ *i* ≤*m*} denote the sets of all circular tracks and all sensors in *M*, respectively. Let *S_i_* denote the set of the sensors located in track *k_i_* and |*S_i_*| denote the number of sensors belonging to set *S_i_*.

All sensors execute the sensing task and then periodically report their readings to the mobile sink in every time period *t*. To collect the readings generated by all sensors, the time duration for the mobile sink to traverse each track for one sweep repetition is not more than *t*. Therefore, the mobile sink will move along different tracks with different velocities. Moreover, when a mobile sink moves along the track *k_i_*, the track *k_i_* is called the *data collection track k_collect_*. To concentrate our discussion on constructing a movement trajectory for the mobile sink such that the data-relaying workloads of all sensors can be totally balanced, this paper assumes that any sensor *s_j_*∉*S_i_* delivers its reading to the sensor *s_k_* ∈ *S_i_* in a multi-hop manner by applying the existing routing protocol [12–15]. After that, sensor *s_k_* forwards its own and the received readings to the mobile sink when the sink passes through its transmission range. Based on this rule, once a mobile sink completes the movement of one sweep repetition on track *k_collect_*, it can collect all readings generated by all sensors in *M*.

### 2.2. Notations

This paper aims to construct a data collection trajectory along which the mobile sink can collect all readings generated by all sensors while the data-relaying workloads of all sensors can be balanced. To achieve this purpose, the mobile sink traverses different tracks for different number of sweep repetitions to collect data. For the ease of presentation, we first introduce some notations.

Let $J_{i}=({\text{x}}_{n}^{i},x_{n-1}^{i},\ldots ,x_{1}^{i})$ denote the *regular trajectory i* that a mobile sink initially traverses, track *k_n_* for ${\text{x}}_{n}^{i}$ repetitions of sweeps and then traverses track *k_n_*_-1_ for $x_{n-1}^{i}$ repetitions of sweeps and so on. After traversing track *k*_1_ for $x_{1}^{i}$ repetitions of sweeps, the mobile sink is said to move along the trajectory *J_i_* in one round. Let $e_{i,k}^{\text{com}}$ denote the energy consumption of sensor *s_i_* when the mobile sink completes the movement of trajectory *J_k_* in one round. The trajectory *J_k_* is called an *energy-balanced trajectory*, noted as $J_{k}^{EB-T}$, if $e_{a,k}^{\text{com}}=e_{b,k}^{\text{com}}$ holds, where. ∀ *s_a_, s_b_* ∈ *S*. Let *l_j_* = 2π(2*jr*−*r*) denote the movement length of mobile sink moving along track *k_j_* for one repetition of sweep. Let $|J_{i}|=\sum_{j=1}^{n}{\text{x}}_{j}^{i}l_{j}$ denote the total movement length of mobile sink moving along the trajectory $J_{i}=({\text{x}}_{n}^{i},x_{n-1}^{i},\ldots ,x_{1}^{i})$ in one round. Let *J^EB-T^* denote the set of all energy-balanced trajectories. An energy-balanced trajectory $J_{i}^{EB-T}$ is referred to as the *energy-balanced trajectory with minimal length*, noted as $J_{\min}^{EB-T}$, if it satisfies the Equation (1):

(1) $$\left|J_{i}^{EB-T}\right|=\min\left(\left|J_{k}^{EB-T}\right|\right),\forall J_{k}^{EB-T}\in J^{EB-T}$$

For instance, consider a circular monitoring region which is geographically partitioned into three circular tracks *k*_3_, *k*_2_, and *k*_1_. The*l*_3_, *l*_2_, and *l*_1_ are 10*πr*, 6*πr*, and 2*πr*, respectively. A regular trajectory *J_a_*=(5, 2, 1) represents that mobile sink initially traverses track *k*_3_ for five sweep repetitions and then traverses track *k*_2_ for two sweep repetitions and finally traverses track *k*_1_ for one sweep repetition. If trajectory *J_a_* is an energy-balanced trajectory, this indicates that all sensors will be energy-balanced when the mobile sink completes the movement of trajectory *J_a_* in each round. Furthermore, since *J_a_* =(5, 2, 1) is an energy-balanced trajectory, trajectory *J_b_* =(5*i*, 2*i, i*) must be the other energy-balanced trajectories, where ∀ *i* ∈ *N*. Obviously, as shown in Equations (2) and (3), the value of | *J_a_*| must be less than or equal to those of | *J_b_*| and therefore trajectory *J_a_* is denoted as $J_{\min}^{EB-T}$. Some additional notations are summarized in Table 1.

(2) $$|J_{a}|=5\times 10\pi r+2\times 6\pi r+1\times 2\pi r=64\pi r$$

(3) $$|J_{b}|=5i\times 10\pi r+2i\times 6\pi r+i\times 2\pi r=64i\pi r,\forall i\in N$$

### 2.3. Problem Formulation

The major objective of this paper is to construct a trajectory $J_{\min}^{EB-T}=(x_{n}^{\min},x_{n-1}^{\min},\ldots ,x_{1}^{\min})$ for a mobile sink to collect data from each sensor. The design of the mobile sink's trajectory should meet the energy-balanced requirement that all sensors are energy-balanced when the mobile sink completes the movement of trajectory $J_{\min}^{EB-T}$ in each round. The following shows the problem formulations of our work.

The problem considered in this paper can be formulated as an integer linear programming labeled from Equations (6) to (11). Let *e_i,j_* denote the energy consumption required for sensor *s_j_* after the mobile sink has traversed track *k_i_* for one sweep repetition. Let $e_{j.\min}^{\text{com}}$ denote the total energy consumption required for sensor *s_j_* when the mobile sink completes the movement of trajectory $J_{\min}^{EB-T}$ in each round. The value of $e_{j.\min}^{\text{com}}$ can be calculated by Equation (4):

(4) $$e_{j.\min}^{\text{com}}=e_{1,j}x_{1}^{\min}+e_{2,j}x_{2}^{\min}+\ldots +e_{(n-1),j}x_{n-1}^{\min}+e_{n,j}x_{n}^{\min}=\sum_{i=1}^{n}e_{i,j}x_{i}^{\min}$$

To meet the energy-balanced requirement, an energy-balanced index $f(e_{1.\min}^{\text{com}},e_{2.\min}^{\text{com}},\ldots ,e_{m.\min}^{\text{com}})$, which is defined according to Jain's Fairness Index [16], is used to measure the degree of energy balancing, where *m* denotes the number of sensors in *M*. The energy-balanced index $f(e_{1.\min}^{\text{com}},e_{2.\min}^{\text{com}},\ldots ,e_{m.\min}^{\text{com}})$ that is normalized between 0 and 1 can be formulated by Equation (5):

(5) $$f\left(e_{1.\min}^{\text{com}},e_{2.\min}^{\text{com}},\ldots ,e_{m.\min}^{\text{com}}\right)=\frac{{\left(\sum_{i=1}^{m}e_{i.\min}^{\text{com}}\right)}^{2}}{m\times \sum_{i=1}^{m}{\left(e_{i.\min}^{\text{com}}\right)}^{2}}$$

In case that all $e_{j.\min}^{\text{com}}$ have the same value where ∀*s_j_* ∈ *s*, the result of the fairness index equals to 1, which is the optimal value. An energy-balanced index of a mechanism approaching 1 indicates that the mechanism provides better fairness in terms of energy balancing. Therefore, as shown in objective Function (6), this paper aims at maximizing the energy-balanced index $f(e_{1.\min}^{\text{com}},e_{2.\min}^{\text{com}},\ldots ,e_{m.\min}^{\text{com}})$ while satisfying Constraint (7) to Constraint (11):

(6) $$\text{Maximize}\;f\left(e_{1.\min}^{\text{com}},e_{2.\min}^{\text{com}},\ldots ,e_{m.\min}^{\text{com}}\right)$$

To make the sensors consume less energy when the mobile sink completes the movement of trajectory $J_{\min}^{EB-T}$ in each round, the number of sweep repetitions for a mobile sink moving along each track should be minimized. Constraint (7) shows this requirement:

(7) $$\text{minimize}\sum_{\forall k_{i}\in K}y_{i,b}$$

In the environmental monitoring application, sensors only need to report their readings periodically to the sink instead of reporting data frequently. Hence, Constraint (8) ensures that each sensor executes the sensing task and then periodically generates a packet to the mobile sink in each time period *t*:

(8) $${\text{g}}_{i}^{t}=1,\;\forall s_{i}\in S$$

Furthermore, Constraints (9) and (10) give the upper and lower bounds of the time duration for a mobile sink collecting data along any track *k_collect_* = *k_i_* for one sweep repetition, respectively. Recall that all sensors execute the sensing task and then periodically report their readings to the mobile sink in every time period *t*. If the *t*^min^ is greater than *t*, obviously, the mobile sink cannot successfully collect all readings in every time period *t*. To guarantee that the readings of all sensors can be completely collected by the mobile sink, the *t*^min^ cannot exceed *t*. That is to say, the upper bound on the time duration of the mobile sink collecting data is *t*. Constraint (9) reflects this requirement:

(9) $$t^{\min}\leq t$$

Contrarily, Constraint (10) shows the lower bound of the time duration for mobile sink completing the movement of track *k_collect_* = *k_i_* for one sweep repetition. Let *c_b,j_* denote the transmission rate for sensor *s_j_* transmitting data to mobile sink *b*, where *s_j_* is located in track *k_i_*. According to the Shannon's Theorem [17], the term *c_b,j_* can be formulated as:

$$c_{b,j}=B\times \log_{2}\left(1+{\text{SNR}}_{dB}\right)$$

where *B* is the bandwidth of the channel and *SNR_dB_* is the signal-to-noise ratio of the communication signal to the Gaussian noise interference. Recall that each sensor periodically generates a packet in each time period *t*. The total amount of data generated by each sensor in every time period *t* is *ρ*. That is, the total amount of data generated by all sensors in every time period *t* is *ρ^m^*, where *m* denotes the number of sensors over *M*. Let $t_{i}^{\text{rec}}$ denote the time duration required for the mobile sink receiving *ρ^m^* data in track *k_collect_* = *k_i_*. Because the average data transmission rate $\bar{C_{i}}$ in track *k_collect_* = *k_i_* can be measured by:

$$\bar{C_{i}}=\frac{1}{\left|S_{i}\right|}\sum_{\forall s_{j}\in S_{i}}c_{b,j},$$

the $t_{i}^{\text{rec}}$ can be simply calculated by:

$$t_{i}^{\text{rec}}=\frac{\rho m}{\bar{C_{i}}}.$$

To ensure that the mobile sink has sufficient time to successfully receive all data generated by all sensors, the *t*^min^ should be greater than or equal to $t_{i}^{\text{rec}}$. Hence, the lower bound on the time duration of the mobile sink collecting data is $t_{i}^{\text{rec}}$. Constraint (10) reflects this requirement:

(10) $$t^{\min}\geq t_{i}^{\text{rec}},\;\forall k_{i}\in K$$

Finally, Constraint (11) gives the flow constraint which guarantees that the total amount of packets transmitted by each sensor equals the packets received from all its neighbors plus the packet generated by itself:

(11) $${\text{z}}_{i}^{f}=\sum_{\forall s_{j}\in N\left(s_{i}\right)}z_{i,j}^{r}+{\text{g}}_{i}^{t},\forall s_{i},s_{j}\in S,i\neq j$$

## 3. The Proposed Energy-Balanced Data Collection (EBDC) Mechanism

This section presents the details of the proposed *EBDC* mechanism which is executed by the mobile sink for constructing the trajectory $J_{\min}^{EB-T}$. At a conceptual level, the *EBDC* mechanism is composed of three major phases: *Initialization Phase, Energy Estimation Phase*, and *Trajectory Construction Phase*. In the *Initialization Phase*, the number of sensors |*S_i_*| in each track *k_i_* will be evaluated while the *Energy Estimation Phase* mainly measures the energy consumption of each sensor when the mobile sink traverses any track for one sweep repetition. In the *Trajectory Construction Phase*, the trajectory $J_{\min}^{EB-T}$ can be planned by the information obtained in the previous two phases. These three phases are executed by the mobile sink. After determining the trajectory $J_{\min}^{EB-T}$, the mobile sink will flood its movement plan, including the movement velocity in each track, the number of sweep repetitions in each track, the starting location and the starting time, to all sensors in the monitoring region. Each sensor can therefore derive the track *k_collect_* the mobile sink is currently visiting. The following presents the details of the three phases.

### 3.1. Initialization Phase

Assume that the mobile sink is moving along the track *k_i_*. Each sensor, say *s_a_*, will send its reading to the closest sensor, say *s_b_*, in the track *k_i_* in a multi-hop manner. Afterward, sensor *s_b_* subsequently relays the reading to the mobile sink when the mobile sink passes through its transmission range. As a result, for any track *k_j_*, all sensors can equally share the data-relaying workloads. To evaluate the workload of each sensor in any track *k_j_*, this phase initially evaluates the number of sensors located in any track *k_j_*.

**Figure 3.**
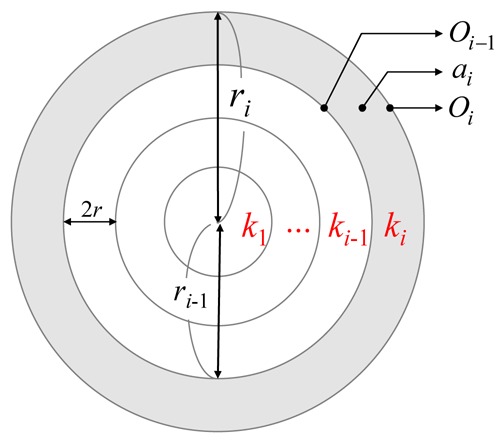
The number of sensors in each track can be evaluated in the *Initialization Phase*.

As shown in Figure 3, let *r_i_* denote the distance between the outside boundary of track *k_i_* and the center of *M*. Recall that the thickness of each track is a constant value 2*r*. The *r_i_* can be represented by notations *i* and *r*, as shown in Equation (12):

(12) $$r_{i}=i\times 2r$$

Let *O_i_* and *a_i_* denote the area sizes of a circle with radius *r_i_* and track *k_i_*, respectively. As shown in Figure 3, the value of *a_i_* can be derived by Equation (13):

(13) $$a_{i}=O_{i}-O_{i-1}=\pi \left(r_{i}^{2}-r_{i-1}^{2}\right)$$

Let *S_i_* denote the set of sensors located in track *k_i_*, and let |*S_i_*| denote the number of sensors belonging to set *S_i_*. For a given WSN with network density *d*, Equation (14) evaluates the value of |*S_i_*| where *n* denotes the total number of tracks:

(14) $$\left|S_{i}\right|=d\times a_{i},\text{where}\;1\leq i\leq n$$

In this phase, the number of sensors |*S_i_*| in each track *k_i_* is evaluated. After completing the executions of this phase, the mobile sink performs the *Energy Estimation Phase*. The following subsection describes the details of *Energy Estimation Phase*.

### 3.2. Energy Estimation Phase

This phase aims to evaluate the energy consumption of each sensor. For the ease of presentation, the following initially introduces several notations.

Let *k_i_ δ k_j_* denote the relative location relation of tracks *k_i_* and *k_j_* where *δ* ∈ {<, >, =}. The value of *δ* is ‘<’, ‘=’, or ‘>’ if the value of (*O_i_– O_j_*) is less than, equal to, or greater than zero. The notation *e_iδj_* denotes the energy consumption required for each sensor located in track *k_j_* when the mobile sink completes the movement of track *k_collect_* = *k_i_* for one sweep repetition.

**Figure 4.**
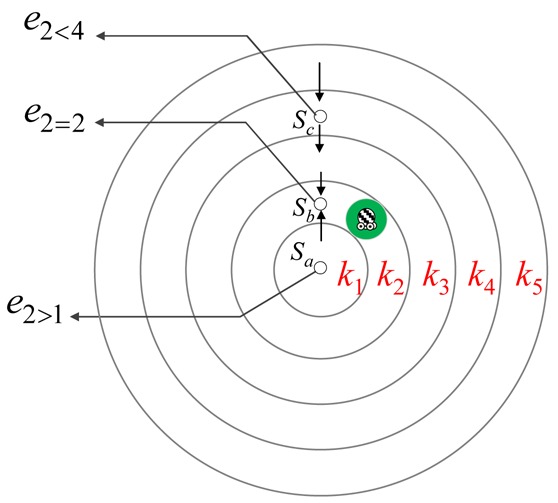
Sensors located in different tracks have different energy consumptions.

Figure 4 depicts an example which illustrates the observation on *e_iδj_* for different values of *δ*. Let sensors *s_a_, s_b_*, and *s_c_* be located in tracks *k*_1_, *k*_2_, and *k*_4_, respectively. Assume that the mobile sink has already completed the movement of track *k*_2_ for one sweep repetition. Since sensor *s_a_* is located in track *k*_1_, the overall workload of *s_a_* is to send its reading to its neighbor in track *k*_2_. On the contrary, sensor *s_c_* not only sends its reading to its neighbor in track *k*_3_ but also needs to relay the data received from the other sensors located in the outer tracks. As a result, we have *e*_2_< 4 > *e*_2_ > 1. Furthermore, the workload of sensor *s_b_* is larger than that of sensors *s_a_* and *s_c_* since *s_b_* not only sends its reading to the mobile sink but also relays data from all tracks other than track *k*_2_ to mobile sink. Consequently, the relations *e*_2_ = 2 > *e*_2_ > 1 and *e*_2_ = 2 > *e*_2_ < 4 hold. According to this observation, we conclude that sensors located in different tracks have different energy consumptions.

Let sensor *s* be located in track *k_y_* and mobile sink has already completed the movement of track *k_x_* for one sweep repetition. Based on the relation *δ*, the energy consumption *e_xδy_* of sensor *s* is discussed for the following three cases.

#### Case 1

*k_x_*> *k_y_*

Let *S_y_* denote the set of sensors located in track *k_y_* and let |*S_y_*| denote the number of sensors belonging to set *S_y_*. Let *p* be the number of packets generated by each sensor in each time period *t*. Let *P_y_^x>y^* represent the total number of packets delivered by all sensors located in the track *k_y_*, for all *y* < *x*. The value of *P_y_^x>y^* can be measured by Equation (15):

(15) $$P_{y}^{x>y}=\sum_{l=1}^{y}\left|S_{l}\right|\times p$$

Let *e_unit_* denote the energy consumption required for each sensor to transmit one packet to its neighbor. Let *E*_*x* > *y*_ denote the total energy consumption required for all sensors located in track *k_y_* when the mobile sink completes the movement of track *k_x_* one sweep repetition, for all *y* < *x*. The value of *E_x_*_>_*_y_* can be calculated by Equation (16):

(16) $$E_{x>y}=P_{y}^{x>y}\times e_{\text{unit}}$$

Consequently, the value of *e_x>y_* can be evaluated by Equation (17):

(17) $$e_{x>y}=E_{x>y}/\left|S_{y}\right|$$

#### Case 2

*k_x_* < *k_y_*

Let *P_y_^x<y^* denote the total number of packets delivered by all sensors located in track *k_y_*, for all *y* > *x*. The value of *P_y_^x<y^* can be measured by Equation (18):

(18) $$P_{y}^{x<y}=\sum_{l=y}^{n}\left|S_{l}\right|\times p$$

Let *E_x_*_<_*_y_* denote the total energy consumption required for all sensors located in track *k_y_* when the mobile sink completes the movement of track *k_x_* for one sweep repetition, for all *y* > *x*. The value of *E_x_*_<_*_y_* can be calculated by Equation (19):

(19) $$E_{x<y}=P_{y}^{x<y}\times e_{\text{unit}}$$

Hence, the value of *e_x<y_* can be evaluated by Equation (20):

(20) $$e_{x<y}=E_{x<y}/\left|S_{y}\right|$$

#### Case 3

*k_x_* = *k_y_*

Let *P_y_^x = y^* denote the total number of packets delivered by all sensors located in track *k_y_*, for all *y* = *x*. The value of *P_y_^x=y^* can be measured by Equation (21):

(21) $$P_{y}^{x=y}=\sum_{l=1}^{n}\left|S_{l}\right|\times p$$

Let *E*_*x* = *y*_ denote the total energy consumption required for all sensors located in track *k_y_* when the mobile sink completes the movement of track *k_x_* for one sweep repetition, for all *y* = *x*. The value of *E*_*x* = *y*_ can be calculated by Equation (22):

(22) $$E_{x=y}=P_{y}^{x=y}\times e_{\text{unit}}$$

As a result, the value of *e_x = y_* can be evaluated according to Expression (23):

(23) $$e_{x=y}=E_{x=y}/\left|S_{y}\right|$$

Table 2 summarizes the energy consumption *e_xδy_* of sensor *s* which is located in track *k_y_*.

### 3.3. Trajectory Construction Phase

In this phase, the trajectory $J_{\min}^{EB-T}$ of the mobile sink will be scheduled using the information obtained in the previous two phases, such that the energy consumptions of all sensors can be totally balanced.

Let $e_{j}^{\text{total}}$ denote the total energy consumption required for any sensor belonging to set *S_j_* when the mobile sink moves one round along the trajectory $J_{\min}^{EB-T}=(x_{n}^{\min},x_{n-1}^{\min},\ldots ,x_{1}^{\min})$. Recall that $x_{i}^{\min}$ denotes the number of sweep repetitions of that mobile sink as it moves along track *k_i_*. The $e_{j}^{\text{total}}$ can be derived by Equation (24):

(24) $$e_{j}^{\text{total}}=\sum_{i=1}^{n}e_{i\delta j}x_{i}^{\min},\;\text{where}\;\delta \in \left\{>,<,=\right\}$$

As shown in objective function (6), the goal of this paper is to maximize the energy-balanced index $f(e_{1.\min}^{\text{com}},e_{2.\min}^{\text{com}},\ldots ,e_{m.\min}^{\text{com}})$. To accomplish this, the energy consumptions of any two sensors should be identical when the mobile sink completes the movement of trajectory $J_{\min}^{EB-T}$ for one round. Consequently, according to Equation (24), we can obtain Equation (25):

(25) $$\left[\begin{array}{cccc} e_{1=1} & e_{2>1} & \ldots & e_{n>1}\\ e_{1<2} & e_{2=2} & \ldots & e_{n>2}\\ \vdots & \vdots & \ddots & \vdots \\ e_{1<n} & e_{2<n} & \cdot \cdot \cdot & e_{n=n} \end{array}\right]\left[\begin{array}{c} x_{1}^{\min}\\ x_{2}^{\min}\\ \vdots \\ x_{n}^{\min} \end{array}\right]=\left[\begin{array}{c} e_{1}^{\text{total}}\\ e_{2}^{\text{total}}\\ \vdots \\ e_{n}^{\text{total}} \end{array}\right]=\left[\begin{array}{c} e_{1}^{\text{total}}\\ e_{1}^{\text{total}}\\ \vdots \\ e_{1}^{\text{total}} \end{array}\right]$$

Equation (26) further derives the value of each variable $x_{i}^{\min}$. To satisfy Constraint (7), the set of the smallest solutions to Equation (26) should be selected:

(26) $$\left[\begin{array}{c} x_{1}^{\min}\\ x_{2}^{\min}\\ \vdots \\ x_{n}^{\min} \end{array}\right]={\left[\begin{array}{cccc} e_{1=1} & e_{2>1} & \ldots & e_{n>1}\\ e_{1<2} & e_{2=2} & \ldots & e_{n>2}\\ \vdots & \vdots & \ddots & \vdots \\ e_{1<n} & e_{2<n} & \cdot \cdot \cdot & e_{n=n} \end{array}\right]}^{-1}\left[\begin{array}{c} e_{1}^{\text{total}}\\ e_{1}^{\text{total}}\\ \vdots \\ e_{1}^{\text{total}} \end{array}\right]$$

However, the set of the smallest solutions derived by Equation (26) might not be an integer solution. If this is the case, for each $x_{i}^{\min}$, we select the integer which is the closest integer to $x_{i}^{\min}$ as the number of sweep repetitions of the mobile sink moving along track *k_i_*. Otherwise, the mobile sink traverses track *k_i_* for $x_{i}^{\min}$ sweep repetitions. As a result, the constructed trajectory $J_{\min}^{EB-T}$ likely achieves the objective Function (6) when the mobile sink completes the movement of trajectory $J_{\min}^{EB-T}$ in each round.

For example, assume that there are three tracks *k*_3_, *k*_2_, and *k*_1_. If $x_{3}^{\min}=5.3$, $x_{2}^{\min}=1.7$, and $x_{1}^{\min}=0.8$ are derived from Equation (26), the numbers of sweep repetitions for a mobile sink moving along tracks *k*_3_, *k*_2_, and *k*_1_ are 5, 2, and 1, respectively. As a result, the mobile sink will initially traverse track *k*_3_ for five sweep repetitions and then traverse track *k*_2_ for two sweep repetitions and finally traverse track *k*_1_ for one sweep repetition.

In summary, a three-phase mechanism is proposed for scheduling the trajectory $J_{\min}^{EB-T}$ for the mobile sink. The data-relaying workloads of all sensors can be totally balanced in each round if the mobile sink moves along the constructed trajectory $J_{\min}^{EB-T}$

## 4. Theoretical Analysis

Section 3 shows the details of the proposed *EBDC* mechanism. By applying *EBDC* mechanism, the data-relaying workloads of all sensors can be totally balanced in an efficient way. In addition to the degree of energy balancing, another crucial factor considered in WSNs is the network lifetime. Herein, the network lifetime is measured by the time interval starting from the time that sensors have been deployed to the time that a coverage hole appears. To verify the performance of the network lifetime, this section further compares the proposed *EBDC* mechanism against the *RMDC* scheme proposed in [11]. The *RMDC* scheme is considered as approach to compare because *RMDC* outperforms related schemes [5–10]. In general, the related data collection schemes can be mainly classfied into *fixed sink schemes* [5–7] and *mobile sink schemes* [8–11]. Unlike the fixed sink schemes [5–7] which are based on a fixed sink, *RMDC* employed a mobile sink to collect data. Hence, the *RMDC* has a better performance than the existing fixed sink schemes [5–7] in terms of network lifetime. Recall that the mobile sink applying the efforts described in [8,9] has to pass through the transmission range of each sensor, thereby leading to a long data collection latency of each sensor. On the contrary, the mobile sink which applies the *RMDC* scheme does not need to pass through the transmission range of each sensor. Therefore, the waiting time for each sensor sending its readings to the mobile sink can be reduced significantly. Furthermore, by applying the *RMDC* scheme, the number of sensors which can directly communicate with the mobile sink is more than that by applying the approach presented in [10]. That is to say, by applying the *RMDC* scheme, the data-relaying workloads can be reduced, prolonging the network lifetime. As a result, the *RMDC* scheme also outperforms the existing mobile sink schemes [8–11].

The considered network environment is a circular monitoring region *M* which has been geographically partitioned into *n* circular tracks. In the proposed *EBDC* mechanism, the trajectory $J_{\min}^{EB-T}=(x_{n}^{\min},x_{n-1}^{\min},\ldots ,x_{1}^{\min})$ is scheduled by the proposed three-phase execution. Let $t_{\text{round}}^{\text{EBDC}}$ denote the time duration of each round. For simplicity and without loss of generality, assume that the time duration for mobile sink traversing each track for one sweep repetition is set to *t*. The value of $t_{\text{round}}^{\text{EBDC}}$ can be evaluated by Equation (27):

(27) $$t_{\text{round}}^{\text{EBDC}}=x_{1}^{\min}t+x_{2}^{\min}t+\ldots +x_{n-1}^{\min}t+x_{n}^{\min}t=t\times \sum_{i=1}^{n}x_{i}^{\min}$$

Since the data-relaying workloads of all sensors can be balanced in each round, to simplify the analysis, the following discusses the energy consumption of the sensor, *s_i_* ∈ *S_n_* where *S_n_* denotes the set of sensors located in the outmost (boundary) track *k_n_*. Let $e_{n}^{\text{total}}$ denote the total energy consumption required for any sensor belonging to set *S_n_* in each round. According to Equation (24), the value of $e_{n}^{\text{total}}$ can be calculated by the Equation (28):

(28) $$e_{n}^{\text{total}}=e_{1<n}x_{1}^{\min}+e_{2<n}x_{2}^{\min}+\ldots +e_{(n-1)<n}x_{n-1}^{\min}+e_{n=n}x_{n}^{\min}$$

Let $e_{l.\text{unit}}^{\text{EBDC}}$ denote the energy consumption required for sensor *s_l_* in each time unit. The $e_{l.\text{unit}}^{\text{EBDC}}$ can be formulated by Equation (29):

(29) $$e_{l.\text{unit}}^{\text{EBDC}}=\frac{e_{n}^{\text{total}}}{t_{\text{round}}^{\text{EBDC}}}=\frac{e_{1<n}x_{1}^{\min}+e_{2<n}x_{2}^{\min}+\ldots +e_{(n-1)<n}x_{n-1}^{\min}+e_{n=n}x_{n}^{\min}}{t\times \sum_{i=1}^{n}x_{i}^{\min}}$$

Since the energy consumption of sensor *s_l_* can be derived by the total energy consumption in track *k_n_* divided by the total number of sensors in track *k_n_*, Equation (30) can be obtained by substituting Equations (20) and (23) into Equation (29):

(30) $$e_{l.\text{unit}}^{\text{EBDC}}=\left(\frac{E_{1<n}x_{1}^{\min}+E_{2<n}x_{2}^{\min}+\ldots +E_{(n-1)<n}x_{n-1}^{\min}+E_{n=n}x_{n}^{\min}}{t\times \sum_{i=1}^{n}x_{i}^{\min}}\right)\left(\frac{1}{\left|S_{n}\right|}\right)$$

Furthermore, the total energy consumption in track *k_n_* can be evaluated by the multiplication of the total number of packets and the energy consumption required for transmitting a packet, Equation (31) can be obtained by substituting Equations (19) and (22) into Equation (30):

(31) $$e_{l.\text{unit}}^{\text{EBDC}}=\left(\frac{P_{n}^{1<n}x_{1}^{\min}+P_{n}^{2<n}x_{2}^{\min}+\ldots +P_{n}^{(n-1)<n}x_{n-1}^{\min}+P_{n}^{n=n}x_{n}^{\min}}{t\times \sum_{i=1}^{n}x_{i}^{\min}}\right)\left(\frac{e_{\text{unit}}}{\left|S_{n}\right|}\right)$$

Since each sensor generates *p* packets in each time period *t*, based on the Equations (18) and (21), the $e_{l.\text{unit}}^{\text{EBDC}}$ can be further derived by Equation (32):

(32) $$\begin{array}{c} e_{l.\text{unit}}^{\text{EBDC}}=\left(\frac{x_{1}^{\min}\sum_{i=n}^{n}\left|S_{i}\right|+x_{2}^{\min}\sum_{i=n}^{n}\left|S_{i}\right|+\ldots +x_{n-1}^{\min}\sum_{i=n}^{n}\left|S_{i}\right|+x_{n}^{\min}\sum_{i=1}^{n}\left|S_{i}\right|}{t\times \sum_{i=1}^{n}x_{i}^{\min}}\right)\left(\frac{p\times e_{\text{unit}}}{\left|S_{n}\right|}\right)\\ =\left(\frac{\left|S_{n}\right|x_{1}^{\min}+\left|S_{n}\right|x_{2}^{\min}+\ldots +\left|S_{n}\right|x_{n-1}^{\min}+x_{n}^{\min}\sum_{i=1}^{n}\left|S_{i}\right|}{t\times \sum_{i=1}^{n}x_{i}^{\min}}\right)\left(\frac{p\times e_{\text{unit}}}{\left|S_{n}\right|}\right)\\ =\left(\frac{\sum_{j=1}^{n-1}x_{j}^{\min}+x_{n}^{\min}\sum_{i=1}^{n-1}\left|S_{i}\right|}{\sum_{j=1}^{n}x_{j}^{\min}}\right)\left(\frac{p\times e_{\text{unit}}}{t}\right) \end{array}$$

Equation (32) indicates that the energy consumption of sensor *s_l_*∈*S_n_* is highly impacted by the parameters, including the number of sweep repetitions performed by the mobile sink and the total number of sensors in each track.

On the other hand, the energy consumption of *RMDC* scheme is analyzed below. Recall that the key idea of *RMDC* scheme is that mobile sink moves along the boundary track of *M* for collecting data generated by all sensors in *M*. For simplicity, we discuss the energy consumption of sensor *s_l_* that is located in the boundary track *k_n_*. To facilitate the analysis, herein, the *round of data collection in RMDC scheme* is initially introduced. As shown in Figure 1, when a mobile sink completes the movement of boundary track *k_n_* one sweep repetition in the clockwise direction, the mobile sink is said to move along the boundary of *M* in one round.

Let *S* denote the set of all sensors in *M*. Let *S_n_* denote the set of boundary sensors, each of which can communicate with mobile sink when the sink passes through its transmission range. Since the mobile sink always moves along the boundary track *k_n_*, only sensors belonging to set *S_n_* are able to play the relay roles to deliver the data generated by the sensors belonging to sets *S* − *S_n_* to mobile sink. Let $P_{\text{round}}^{\text{RMDC}}$ denote the workloads of sensors belonging to set *S_n_* by applying *RMDC* scheme. The value of $P_{\text{round}}^{\text{RMDC}}$ can be evaluated by Equation (33):

(33) $$P_{\text{round}}^{\text{RMDC}}=p\times \sum_{i=1}^{n}\left|S_{i}\right|$$

Let $E_{\text{round}}^{\text{RMDC}}$ denote the total energy consumption required for all sensors belonging to set *S_n_* in each round. The value of $E_{\text{round}}^{\text{RMDC}}$ can be calculated by Equation (34):

(34) $$E_{\text{round}}^{\text{RMDC}}=P_{\text{round}}^{\text{RMDC}}\times e_{\text{unit}}$$

Let $e_{l}^{\text{round}}$ denote the energy consumption of sensor *s_l_* in each round. The value of $e_{l}^{\text{round}}$ can be derived by Equation (35):

(35) $$e_{l}^{\text{round}}=E_{\text{round}}^{\text{RMDC}}/\left|S_{n}\right|$$

Assume that the time duration for mobile sink traversing track *k_n_* for one sweep repetition is also set to *t*. The energy consumption $e_{l.\text{unit}}^{\text{RMDC}}$ required for sensor *s_l_* in each time unit can be estimated by Equation (36):

(36) $$\begin{array}{l} e_{l.\text{unit}}^{\text{RMDC}}=\frac{e_{l}^{\text{round}}}{t}=\frac{E_{\text{round}}^{\text{RMDC}}}{\left|S_{n}\right|\times t}=\frac{P_{\text{round}}^{\text{RMDC}}\times e_{\text{unit}}}{\left|S_{n}\right|\times t}\\ =\frac{p\times \sum_{i=1}^{n}\left|S_{i}\right|\times e_{\text{unit}}}{\left|S_{n}\right|\times t}=\left(\sum_{i=1}^{n-1}\left|S_{i}\right|\right)\left(\frac{p\times e_{\text{unit}}}{t}\right) \end{array}$$

To compare the proposed *EBDC* mechanism with the existing *RMDC* scheme in terms of network lifetime, Equations (32) and (36) are further observed. It is obvious that the relations:

(37) $$\sum_{i=1}^{n-1}\left|S_{i}\right|=\frac{\sum_{i=1}^{n-1}\left|S_{i}\right|\times \sum_{j=1}^{n}x_{j}^{\min}}{\sum_{j=1}^{n}x_{j}^{\min}}>\frac{x_{n}^{\min}\sum_{i=1}^{n-1}\left|S_{i}\right|}{\sum_{j=1}^{n}x_{j}^{\min}}$$

and:

(38) $$\frac{\sum_{j=1}^{n-1}x_{j}^{\min}+x_{n}^{\min}\sum_{i=1}^{n-1}\left|S_{i}\right|}{\sum_{j=1}^{n}x_{j}^{\min}}>\frac{x_{n}^{\min}\sum_{i=1}^{n-1}\left|S_{i}\right|}{\sum_{j=1}^{n}x_{j}^{\min}}$$

hold. For the ease of presentation, let:

$$\begin{array}{l} a=\sum_{i=1}^{n-1}\left|S_{i}\right|,\\ b=\left(\sum_{j=1}^{n-1}x_{j}^{\min}+x_{n}^{\min}\sum_{i=1}^{n-1}\left|S_{i}\right|\right)/\sum_{j=1}^{n}x_{j}^{\min},\text{and}\\ c=\left(x_{n}^{\min}\sum_{i=1}^{n-1}\left|S_{i}\right|\right)/\sum_{j=1}^{n}x_{j}^{\min}. \end{array}$$

Equation (39) can be obtained according to Equations (37) and (38)

(39) $$\begin{array}{c} a-c=\sum_{i=1}^{n-1}\left|S_{i}\right|-\frac{x_{n}^{\min}\sum_{i=1}^{n-1}\left|S_{i}\right|}{\sum_{j=1}^{n}x_{j}^{\min}}\\ =\frac{\sum_{i=1}^{n-1}\left|S_{i}\right|\times \sum_{j=1}^{n}x_{j}^{\min}}{\sum_{j=1}^{n}x_{j}^{\min}}-\frac{x_{n}^{\min}\sum_{i=1}^{n-1}\left|S_{i}\right|}{\sum_{j=1}^{n}x_{j}^{\min}}\\ =\frac{\sum_{i=1}^{n-1}\left|S_{i}\right|\times \sum_{j=1}^{n-1}x_{j}^{\min}}{\sum_{j=1}^{n}x_{j}^{\min}}>\frac{\sum_{j=1}^{n-1}x_{j}^{\min}}{\sum_{j=1}^{n}x_{j}^{\min}}\\ =\frac{\sum_{j=1}^{n-1}x_{j}^{\min}+x_{n}^{\min}\sum_{i=1}^{n-1}\left|S_{i}\right|}{\sum_{j=1}^{n}x_{j}^{\min}}-\frac{x_{n}^{\min}\sum_{i=1}^{n-1}\left|S_{i}\right|}{\sum_{j=1}^{n}x_{j}^{\min}}=b-c \end{array}$$

As a result, we have *a* − *c* > *b* − *c*. That is to say, we have:

(40) $$a>b\Rightarrow \sum_{i=1}^{n-1}\left|S_{i}\right|>\frac{\sum_{j=1}^{n-1}x_{j}^{\min}+x_{n}^{\min}\sum_{i=1}^{n-1}\left|S_{i}\right|}{\sum_{j=1}^{n}x_{j}^{\min}}$$

Equation (40) indicates that the relation holds:

$$\begin{array}{c} e_{l.\text{unit}}^{\text{RMDC}}=\left(\sum_{i=1}^{n-1}\left|S_{i}\right|\right)\left(\frac{p\times e_{\text{unit}}}{t}\right)=a\left(\frac{p\times e_{\text{unit}}}{t}\right)\\ >b\left(\frac{p\times e_{\text{unit}}}{t}\right)=\left(\frac{\sum_{j=1}^{n-1}x_{j}^{\min}+x_{n}^{\min}\sum_{i=1}^{n-1}\left|S_{i}\right|}{\sum_{j=1}^{n}x_{j}^{\min}}\right)\left(\frac{p\times e_{\text{unit}}}{t}\right)=e_{l.\text{unit}}^{\text{EBDC}} \end{array}$$

This implies that the proposed *EBDC* mechanism outperforms the existing *RMDC* scheme in terms of network lifetime.

## 5. Performance Evaluation

This section examines the performance improvement of the proposed *EBDC* mechanism compared with the *Angle-based* approach. Furthermore, the proposed *EBDC* mechanism is also compared with the existing approaches proposed by studies [6] and [11] which are referred to as *Fixed* and *RMDC*, respectively.

**Figure 5.**
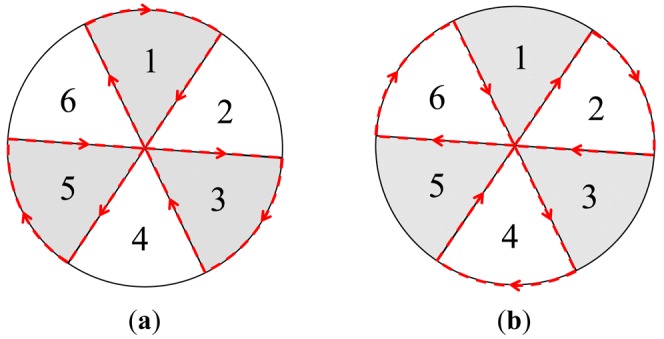
In the odd (even) round, the mobile sink traverses the edges of each fan belonging to odd (even) set one by one in an increasing order of fan number. (**a**) Odd round; (**b**) Even round.

The *Angle-based* approach is a heuristic-based algorithm which initially partitions the circular monitoring region *M* into *f* = 360/*g* fans based on the angle *g*. These fans can be sequentially numbered from 1 to *f* in the clockwise direction. In general, the fans will be classified into two sets: o*dd* and *even* sets. The odd set consists of fans numbered with odd numbers while the even set comprises the remaining fans numbered with even numbers. As shown in Figure 5(a,b), in the odd (even) round, the mobile sink traverses the edges of each fan belonging to the odd (even) set one by one in an increasing order of fan number.

The odd and even rounds will be applied by the mobile sink in turn until the energy of the mobile sink is exhausted. Herein, we assume that all sensors know the traverse rules as mentioned above and are able to estimate the current location of the mobile sink at any given time. The *Fixed* approach employs a fixed sink located at the central point of *M* to collect data while the *RMDC* approach uses a mobile sink moving along the boundary track of *M* to collect the readings as shown in Figure 1.

Table 3 gives the parameters used in our simulation. Each simulation result is obtained from the average of 100 independent runs and the 95% confidence interval is always smaller than 5% of the reported values. The following depicts the results of our performance evaluations.

**Figure 6.**
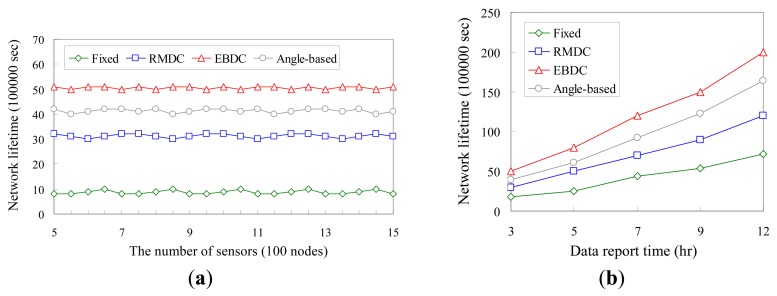
Comparison of *Fixed, Angle-based, RMDC*, and *EBDC* in terms of network lifetime. (**a**) Comparison of four mechanisms in terms of network lifetime by varying the number of sensors; (**b**) Comparison of four mechanisms in terms of network lifetime by varying the data report frequency.

Figure 6(a,b) compare the proposed *EBDC* mechanism with the *Fixed, Angle-based*, and *RMDC* approaches in terms of network lifetime. Herein, the network lifetime is measured by the time interval starting from the time that sensors have been deployed to the time that a coverage hole appears. The four mechanisms are compared by varying the number of sensors and data report time *t* in Figure 6(a,b), respectively.

As shown in Figure 6(a), since there is no sleep-wake scheduling mechanism applied to the WSN, all sensors should keep working on sensing and communication. Thus, the time that the first coverage hole appears does not change a lot in the four approaches compared. As a result, the network lifetimes of *Fixed, Angle-based, RMDC*, and the proposed *EBDC* approaches maintain constant curves. On the contrary, as shown in Figure 6(b), the network lifetimes of *Fixed, Angle-based, RMDC*, and the proposed *EBDC* approaches increase with the data report time *t*. This is because the lower value of data report time means that all sensors will report their readings more frequently, resulting in poor performances in terms of network lifetime.

Moreover, as shown in Figure 6(a,b), in the *Fixed* approach, the sink is fixed and thus the data-relaying workloads totally concentrate on a small number of sensors, leading to a poor network lifetime. As a result, the network lifetime of *Fixed* approach is much shorter than those of the *Angle-based, RMDC*, and *EBDC* approaches. In the *RMDC* approach, since the mobile sink always moves along the outmost (boundary) track, the number of sensors which can be visited by the mobile sink is smaller than those of *Angle-based* and *EBDC* approaches. Hence, the network lifetime of the *RMDC* approach is shorter than those of *Angle-based* and *EBDC* approaches. By applying the *Angle-based* approach, the mobile sink traverses the edges of each fan belonging to odd or even sets one by one, as shown in Figure 5(a,b), respectively. Therefore, the number of sensors which can be visited by the mobile sink is obviously larger than that of *RMDC* approach. Applying the proposed *EBDC* approach, the mobile sink moves with a well established schedule and hence the data-relaying workloads are shared by all sensors. Consequently, the performance of *Angle-based* is worse than *EBDC*. In general, as shown in Figure 6(a), the average network lifetime of the proposed *EBDC* mechanism is approximately five times longer than that of the *Fixed* approach, 1.6 times longer than that of the *RMDC* scheme, and 1.2 times longer than that of the *Angle-based* approach. On the other hand, as shown in Figure 6(b), the average network lifetime of the proposed *EBDC* mechanism is approximately three times longer than that of the *Fixed* approach, 2.2 times longer than that of the *RMDC* scheme, and 1.2 times longer than that of the *Angle-based* approach.

**Figure 7.**
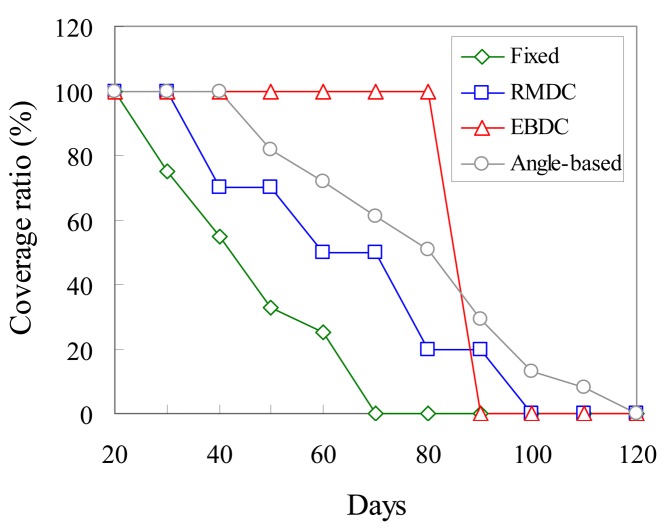
Comparison of *Fixed, Angle-based, RMDC*, and *EBDC* in terms of coverage ratio *σ*.

Figure 7 further measures the monitoring quality when the coverage hole appears. It compares the proposed *EBDC, Angle-based, Fixed*, and *RMDC* approaches in terms of the coverage ratio *σ*. Let *A_cover_* denote the area size which is covered by sensors in the monitoring region. Let *A_M_* denote the area size of the monitoring region. The coverage ratio *σ* can be formulated by the Expression (41):

(41) $$\sigma =A_{\text{cover}}/A_{M}$$

The four approaches have 100% coverage ratio for 20 days starting from the day that the four approaches are applied. Since the data-relaying workload of the *Fixed* approach is totally shared by a small number of sensors, the curve of the *Fixed* approach drops earlier than the curves of the other compared schemes. The coverage ratio of *RMDC* approach is decreased with the elapsed days. In particular, it is interesting that the *RMDC* curve has a stair shape. This is because the mobile sink always moves along the boundary track (track *k_n_*) of the monitoring region. Hence, the sensors located in the boundary track simultaneously exhaust their energies. As a result, the *RMDC* curve drops significantly. After that, the mobile sink will treat track *k_n_*_−1_ as the new boundary track. The coverage ratio of the *Angle-based* approach is also decreased with the elapsed days. However, in the *Angle-based* approach, since the number of sensors which share the data-relaying workloads is larger than that of *RMDC*, the *Angle-based* curve drops slower than *RMDC* curve. In the proposed *EBDC* mechanism, the mobile sink moves along trajectory $J_{\min}^{EB-T}$ to collect data. As a result, the energy consumptions of all sensors can be balanced and thus the *EBDC* curve keeps a constant shape. In general, the proposed *EBDC* mechanism has either 0% or 100% coverage ratios.

**Figure 8.**
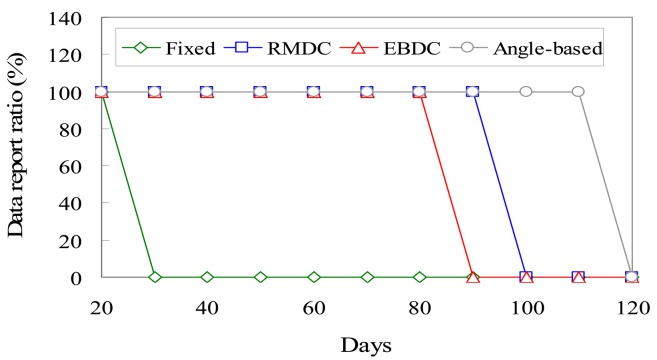
Comparison of *Fixed, Angle-based, RMDC*, and *EBDC* in terms of data report ratio *ξ*.

Figure 8 compares the proposed *EBDC, Angle-based, Fixed*, and *RMDC* approaches in terms of the data report ratio *ξ*. Let *p_success_* denote the number of packets which are successfully forwarded to the sink. Let *P_total_* denote the total number of packets which are generated by all sensors. The data report ratio *ξ* can be formulated by the Expression (42):

(3) $$\xi =p_{\text{success}}/P_{\text{total}}$$

In the *Fixed* approach, the neighboring sensors of the fixed sink will exhaust their energies prior to other sensors. Once these sensors fail, no sensor can directly communicate with the fixed sink, resulting in network partition. As a result, as shown in Figure 8, the *Fixed* curve sharply drops when the neighboring sensors of the fixed sink fail. The *Angle-based, RMDC*, and *EBDC* approaches employ mobile sink to collect data. By applying these three approaches, the readings generated by sensors can always be forwarded to mobile sink since these three schemes maintain the network connectivity using mobile sink. Consequently, the data report ratios of *Angle-based, RMDC*, and *EBDC* keep constant values. However, the proposed *EBDC* mechanism guarantees no coverage holes appeared in the monitoring region while the *Angle-based* and *RMDC* approaches result in coverage holes, as shown in Figure 7.

**Figure 9.**
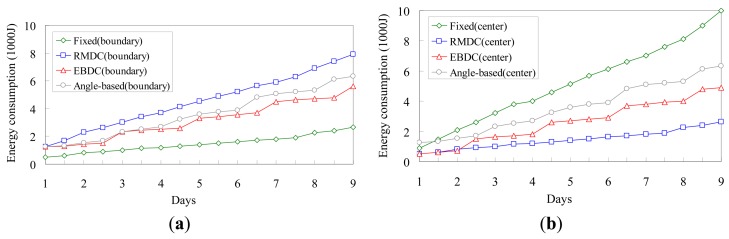
Comparison of *Fixed, Angle-based, RMDC*, and *EBDC* in terms of energy consumption. (**a**) The energy consumption of a randomly selected boundary sensor; (**b**) The energy consumption of a randomly selected central sensor.

Figure 9(a,b) investigate the total energy consumptions required for sensors located in the boundary track (track *k_n_*) and center track (track *k*_1_), respectively. We randomly select a *boundary sensor* and a *central sensor* that are located in the boundary and center tracks, respectively, and then observe their energy consumptions.

The *Fixed* approach deploys a fixed sink located at the central point of the monitoring region. This implies that the selected boundary sensor only needs to deliver its reading to its neighbor without any packet forwarding workloads. Hence, as shown in Figure 9(a), the *Fixed* scheme has better performance than the other three compared approaches. However, as shown in Figure 9(b), the *Fixed* scheme has the worst performance since sensors closer to the fixed sink would have much higher data-relaying workloads. By applying the *RMDC* approach, the mobile sink always moves along the boundary track to collect data. Consequently, the boundary sensor has much higher data-relaying workloads while the central sensor only needs to deliver its reading without any packet forwarding workload. As a result, the *RMDC* has the best performance in Figure 9(b) and the worst performance in Figure 9(a). In the proposed *EBDC* and *Angle-based* approaches, both selected boundary and central sensors will be visited by mobile sink. Therefore, the performances of the proposed *EBDC* and *Angle-based* approaches in Figure 9(a,b) are between the *Fixed* and *RMDC* approaches. In particular, since the proposed *EBDC* mechanism takes into consideration the factor of energy balancing, the performance of *EBDC* is better than that of *Angle-based* scheme, as shown in Figure 9(a,b).

**Figure 10.**
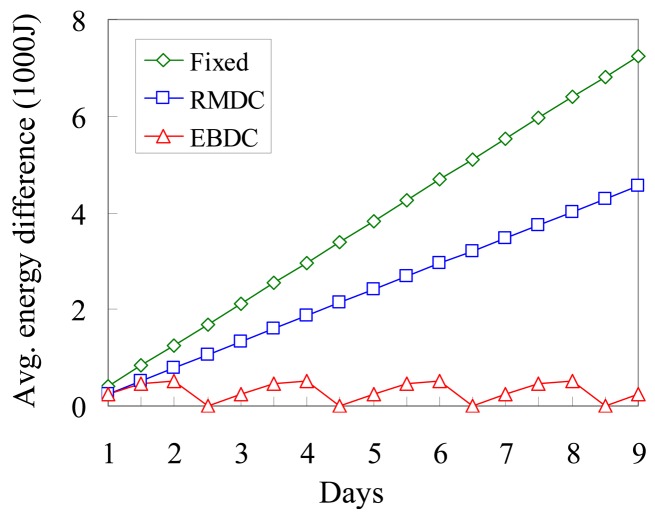
Comparison of *Fixed, RMDC*, and *EBDC* in terms of the degree of energy balancing.

Figure 10 compares the proposed *EBDC, Fixed*, and *RMDC* approaches in terms of the degree of energy balancing. Without loss of generality, we investigate the average energy difference between the central and boundary sensors. The average energy differences of the *Fixed* and *RMDC* approaches are increased with the elapsed days. This is because the two sinks applying the *Fixed* and *RMDC* approaches execute the data collection task in the center and boundary tracks, respectively. The data-relaying workloads of *Fixed* and *RMDC* approaches are hence concentrated on the central and boundary sensors, respectively, leading to an energy-unbalance problem. In the proposed *EBDC* mechanism, the data-relaying workloads of all sensors can be totally balanced in each round. Therefore, as shown in Figure 10, the average energy difference of the proposed *EBDC* mechanism periodically drops to 0 Joule when the mobile sink completes the data collection task in each round. In general, the proposed *EBDC* mechanism outperforms the *Fixed* and *RMDC* schemes in terms of the degree of energy balancing in all cases.

**Figure 11.**
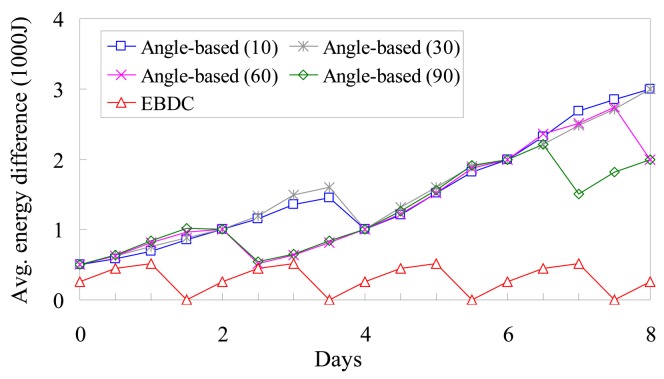
Comparison of *Angle-based* and *EBDC* in terms of the degree of energy balancing.

Figure 11 further compares the proposed *EBDC* mechanism with the *Angle-based* approach in terms of the degree of energy balancing. The fan angle *g* of *Angle-based* approach is set to 10°, 30°, 60°, and 90°. Similar to Figure 10, we investigate the average energy difference between the randomly selected boundary and central sensors. In the *Angle-based* approach, the mobile sink traverses the edges of each fan starting form the center of the monitoring region. As shown in Figure 5(a,b), the central sensor will be visited when mobile sink completes the traverse of the edges of each fan. Therefore, as shown in Figure 11, the three *Angle-based* curves are increased with the elapsed days most of the time. In particular, the three curves drop only when the boundary sensor is visited by mobile sink. However, since the *Angle-based* approach does not consider the factor of energy balancing, the three *Angle-based* curves cannot drop to 0 Joule. On the contrary, the *EBDC* curve periodically drops to 0 Joule when mobile sink completes the data collection task in each round. In general, the proposed *EBDC* mechanism outperforms the *Angle-based* approach in terms of the degree of energy balancing in all cases.

## 6. Conclusions

This paper proposes an *EBDC* mechanism for mobile sinks to collect data generated by all sensors. Initially, the circular monitoring region is geographically partitioned into a number of circular tracks. Then, the mobile sink moves along the scheduled trajectory $J_{\min}^{EB-T}$ for data collection such that the energy consumptions of all sensors can totally be balanced. The proposed *EBDC* mechanism mainly consists of three phases: *Initialization, Energy Estimation*, and *Trajectory Construction Phases*. The *Initialization Phase* evaluates the number of sensors |*S_i_*| in each track *k_i_* while the *Energy Estimation Phase* derives the energy consumption required for each sensor when mobile sink traverses any track for one sweep repetition. Eventually, the *Trajectory Construction Phase* schedules the movement trajectory $J_{\min}^{EB-T}$ for mobile sink. When mobile sink completes the movement of trajectory $J_{\min}^{EB-T}$ in each round, the data-relaying workloads can be totally shared by all sensors. That is to say, the energy consumptions of all sensors can be balanced. Theoretical analysis and performance evaluation reveal that the proposed *EBDC* mechanism outperforms existing approaches in terms of network lifetime and the degree of energy balancing achieved.

## Figures and Tables

**Table 1.** Additional notations.

*y_i,b_*	Number of repetitions of sweeps for mobile sink *b* moving along track *k_i_* when mobile sink completes the movement of the constructed trajectory $J_{\min}^{EB-T}$ in each round.
*t*^min^	Time duration for mobile sink traversing each track one repetition of sweep when it moves along the trajectory $J_{\min}^{EB-T}$.
*t*	A user predefined value.
*ρ*	Size of each packet.
${\text{g}}_{i}^{t}$	Total amount of packets generated by each sensor in every time period *t*.
${\text{z}}_{i}^{f}$	Total amount of packets forwarded by sensor *s_i_* in every time period *t*.
*N*(*s_i_*)	The set of sensor *s_i_*'s neighbors.
$z_{i,j}^{r}$	Total amount of packets received by sensor *s_i_* from its neighbor *s_j_* in every time period *t*.

**Table 2.** The energy consumption of sensor *s*.

**Case**	**Relation**	**Result**
1	*k_x_* > *k_y_*	*e_x>y_* = *E_x>y_*/|*S_y_*|
2	*k_x_* < *k_y_*	*e_x<y_* = *E_x<y_*/|*S_y_*|
3	*k_x_* = *k_y_*	*e_x=y_* = *E_x=y_*/|*S_y_*|

**Table 3.** Simulation parameters.

Network radius *R*	1,500 m
The number of sensors	500∼1,500 nodes
Initial energy of each sensor	10,000 J
Packet transmission cost	0.075 J/s
Packet reception cost	0.030 J/s
Idle cost	0.025 J/s
Data report time *t*	3∼12 h
